# Secretor and non-secretor human milk oligosaccharides differentially modulate immune response in the presence of cow’s milk allergen β-lactoglobulin in an *in vitro* sensitization model

**DOI:** 10.3389/fimmu.2025.1575656

**Published:** 2025-05-09

**Authors:** Anneke H. Hellinga, Marit Zuurveld, Marko Mank, Aletta D. Kraneveld, Johan Garssen, Kennedy Spann, Lars Bode, Linette E. M. Willemsen, Belinda van’t Land

**Affiliations:** ^1^ Center for Translational Immunology, University Medical Center Utrecht, Utrecht, Netherlands; ^2^ Division of Pharmacology, Utrecht Institute for Pharmaceutical Sciences (UIPS), Utrecht University, Utrecht, Netherlands; ^3^ Global Center of Excellence Human Milk Research and Analytics, Danone Global Research and Innovation Center, Utrecht, Netherlands; ^4^ Department of Neuroscience, Faculty of Science, Vrije Universiteit (VU) University, Amsterdam, Netherlands; ^5^ Global Center of Excellence Immunology, Danone Global Research and Innovation Center, Utrecht, Netherlands; ^6^ Department of Pediatrics, Larsson-Rosenquist Foundation Mother-Milk-Infant Center of Research Excellence (MOMI CORE), and the Human Milk Institute (HMI), University of California San Diego, La Jolla, CA, United States

**Keywords:** β-lactoglobulin (BLG), cow’s milk allergy (CMA), early life immune development, human milk oligosaccharides (HMOs), *in vitro* intestinal mucosal immunity, non-secretor, secretor

## Abstract

**Introduction:**

Food allergies, like cow’s milk allergy, significantly impact children, with sensitization often beginning during the first year of life. Human milk oligosaccharides (HMOs) may influence this process, as specific HMOs differentially affect mucosal immune responses *in vitro*. Given the distinct HMO profiles of secretor (Se+) and non-secretor (Se-) milk, we investigate how the full HMO profiles from Se+ and Se- milk affect immune responses in the absence or presence of a cow’s milk allergen.

**Methods:**

Monocyte-derived dendritic cells (moDCs) were exposed to isolated Se+ and Se- pooled HMOs (pHMOs), and subsequently co-cultured with naïve T cells to confirm immune modulation. We compared the type 2-activation capability of several cow’s milk proteins via direct exposure to moDCs or via intestinal epithelial cells (IECs) co-cultured with moDCs. Finally, we studied the effect of pHMOs in the presence of cow’s milk allergen β-lactoglobulin (BLG) (via (IECs)) on moDCs and subsequent T cell response.

**Results:**

Both Se+ and Se- pHMOs dose-dependently activated moDCs, indicated by increased IL8 release and %CD80+ moDCs. Se+ pHMOs tended to increase type 2-associated markers, while also increasing regulatory IL10 release. Se+ pHMOs-pre-exposed moDCs instructed T cells to produce type 2 cytokines like IL13. Se- pHMOs reduced the %CD86+ moDCs but did not drive a type 2 signature in T cells. In the presence of BLG, Se+ pHMOs-pre-exposed moDCs also instructed IL13 release by T cells, while increasing the percentage regulatory T cells. In contrast, co-exposure of BLG with Se- pHMOs only slightly affected moDC phenotype, and these moDCs did not modify T cell phenotypes.

**Conclusions:**

Se+ and Se- pHMOs with BLG differentially affected moDC activation. Se+ pHMO-pre-exposed moDCs induced a type 2- and regulatory-associated T cell phenotype. These data suggest that depending on the secretor status, HMOs differentially modulate immune responsiveness *in vitro*.

## 1 Introduction

The prevalence of food allergies is on the rise globally, affecting up to 10% of European children (1). Cow’s milk, peanut and hen’s egg allergy are among the most common food allergies (2). Although 60-75% of children who are allergic to cow’s milk have overgrown this allergy by age 5 (3), it remains the most prevalent food allergy during the first year of life (4). Major cow’s milk allergens include whey protein β-lactoglobulin (BLG) and caseins αs1- and αs2-casein (5). With low levels of these cow’s milk allergens in human milk, and most common alternatives to breastfeeding are cow’s milk based formula, the burdensome symptoms can occur very early in life possibly affecting nutritional status of infants, as well as the quality of life of both infants and their parents (6, 7).

Allergic sensitization is a result of impaired tolerance and a T helper (Th) 2-driven immune response specific for an allergen. Upon exposure, allergens may cause epithelial cells to produce alarmins (8), priming underlying dendritic cells (DCs) toward a type 2 response. This type 2 priming in DCs is characterized by secretion of e.g. C-C motif chemokine ligand 22 (CCL22) and expression of costimulatory molecules and receptors for alarmins, such as thymic stromal lymphopoietin (TSLP) and interleukin (IL) 33 (9, 10). Type 2-primed DCs present their captured allergen in the lymph nodes to naïve T cells, stimulating polarization into Th2 cells rather than regulatory cells or Th1 cells. Th2 cells are characterized by secretion of IL4 and IL13 and expression of prostaglandin D2 receptor 2 (or chemoattractant receptor homologous molecule expressed on T helper type 2 cells (CRTH2)) (11). Allergen-specific Th2 cells promote isotype switching of allergen-specific B cells to produce allergen-specific IgE. Upon re-exposure, degranulation of mast cells and basophils lead to local symptoms, possibly expanding to systemic responses (12).

Several approaches to prevent sensitization during early life have been studied (13). Breastfeeding is the universal gold standard for infant feeding (14). Although several studies found breastfeeding to protect against allergic diseases in general, meta-analysis and systematic reviews on cohort and cross-sectional studies do not conclude on an association between breastfeeding and food allergies (15, 16). Heterogeneity in outcome assessment, as well as the heterogeneity in human milk composition between individuals and over the course of lactation, complicates comparison of results. In addition, performing high quality studies, such as randomized controlled trials, is challenging due to ethical and practical reasons (15, 16). *In vitro* and *in vivo* studies are, therefore, utilized to study the association between and potential mechanisms underlying human milk components, such as human milk oligosaccharides (HMOs) and atopic diseases.

Human milk contains a variety of components, of which HMOs are the third most abundant, after lactose and lipids. More than 150 different and distinct HMO structures have been identified today (17–19). The HMO composition in human milk is highly variable. The majority of variation is attributed to polymorphisms in the fucosyltransferase 2 gene (FUT2), as it affects fucosylation of HMOs (20, 21). An active FUT2 gene allows fucosylation of HMO core-structures via an α1,2-glycosidic linkage, defining secretors (Se+). Se+ milk is characterized by high levels of 2’-fucosyllactose (2’-FL) and lacto-*N*-fucopentaose I (LNFP I). Non-secretors (Se-) are defined by an inactive FUT2 enzyme and a (near-)absence of α1,2-fucosylated HMOs like 2’-FL and LNFP I (21–25).

Some studies investigated the association of maternal secretor status with atopy development of breastfed infants. Recently, a relatively large observational study, including human milk samples from 980 breastfeeding women, showed that high concentrations of fucosylated HMOs 2’-FL and difucosyllacto-*N*-hexaose, as well as total HMOs and total HMO-bound fucose were associated with reduced prevalence of recurrent wheezing in high-risk children (21). Additionally, lower incidence of IgE-associated eczema at two years of age was observed for previously breastfed infants who received Se+ milk. However, this was only true for infants who were born via cesarean section (26). Another observational cohort did not find a significant difference for the risk of developing atopic dermatitis in the first two years of life between infants receiving Se+ or Se- milk, although way of delivery was not considered in their analysis (27). These yet inconclusive findings highlight the need to further study the role of HMOs and secretor status in the risk of atopy development, including food allergies, in an *in vitro* setting to understand differences in immune response to allergens and the potential underlying mechanisms.

HMOs have bioactive functions ranging from acting as prebiotics and supporting the epithelial barrier to directly interacting with immune cells (28). Although HMOs have been detected in blood, stool and urine of breastfed infants (29–32), HMOs also interact locally in the intestine with the mucosal immune system (29–32). An *in vitro* study showed that pooled HMOs (pHMOs) induced semi-maturation of monocyte-derived DCs (moDCs), characterized by increased expression of PD-L1 and secretion of among others IL10. Naïve T cells co-cultured with pHMO-pre-exposed moDCs led to an increase of CD25+/FoxP3+ regulatory T cells (Tregs) and increased IL10 secretion. In addition, although not significant, the ratio of Th1 (Tbet+) to Th2 (GATA3+) cell ratio tended to favor Th1 cells (33). Another study investigated the effect of HMOs 2’-FL and 3-FL in a sensitization co-culture model including intestinal epithelial cells, moDCs and T cells subsequently. T cells co-cultured with moDCs primed with IECs pre-exposed to ovalbumin (OVA) and 2’-FL induced an inflammatory and regulatory response, characterized by increased secretion of IL13, IFNγ, IL17 and IL10, compared to OVA exposure only. In contrast, T cells co-cultured with moDCs primed with IECs pre-exposed to OVA and 3-FL, decreased the secretion of IL13 compared to OVA only, implying a shift toward IL17A and IL10 (34). These results indicate that HMOs differentially modulate the immune response, although generally, individual HMOs or mixtures are thought to skew the immune response toward a type 1 and regulatory response (28).

Although several studies have explored immunomodulatory properties of individual, synthesized HMOs, in the perspective of breastfeeding, the effect of the complete HMO profile is most relevant. As the HMO profile is fundamentally different between milk from Se+ and Se- mothers, we studied the influence of Se+ and Se- pHMOs, respectively, on moDC maturation and differentiation, with and without interference of intestinal epithelium. We compared the sensitization capacity of several relevant food allergens on epithelial cells and/or moDCs. Lastly, we studied the immunomodulatory effects of Se+ and Se- pHMOs in the light of allergic sensitization on BLG-exposed (IEC-)moDCs and subsequent effect on co-cultured allogenic naïve CD4+ T cells.

## 2 Materials and methods

### 2.1 Pooled human milk oligosaccharides (pHMOs)

Human milk was collected as part of UC San Diego’s Human Milk Institute human milk donation program. Healthy donors provided milk in excess of their own baby’s needs. Milk was screened for secretor status by fluorescence high-performance liquid chromatography (HPLC-FL). Secretor milk from 13 donors and non-secretor milk from 9 donors were pooled separately to generate a secretor and a non-secretor pool. The full Se+ and Se- HMO profile was isolated from these separate donor human milk pools as previously described (35). After centrifugation, the lipid layer was removed, and proteins were precipitated from the aqueous phase by addition of ice-cold ethanol and subsequent centrifugation. Ethanol was removed from the HMO-containing supernatant by roto-evaporation. Lactose and salts were removed by gel filtration chromatography over a BioRad P2 column (100 cm x 316 mm, Bio-Rad) using a semi-automated fast protein liquid chromatography (FPLC) system. Only pHMOs with less than 2% lactose were used for experiments.

Endotoxin levels were assessed in a 10%w/v pHMO in sterile PBS solution as >10^4^ng/mL by a LAL assay according to manufacturer’s protocol (Charles River, USA), using Endotoxin-Specific Buffer (Charles River, USA). Both samples were further diluted to 5%w/v in endotoxin-free water (Sigma-Aldrich, UK). Endotoxin removal was performed with spin columns according to manufacturer’s protocol (Thermo Fisher Scientific, USA). After endotoxin removal, Se+ and Se- pHMOs contained 25.2ng/mL and 9.5ng/mL endotoxins, respectively, at the highest concentration (0.5%) used in the (co-)culture experiments. An LPS control of 25ng/mL was included in the experiments. HMO composition was assessed before and after endotoxin removal by high pressure anion exchange chromatography coupled to pulsed amperometric detection (HPAEC-PAD) as previously described (36, 37). Levels of the five most abundant HMOs were quantified before and after endotoxin removal with a 80-95% recovery (**Supplementary Table S1**).

### 2.2 Allergens

Hen’s egg allergen OVA (A5503, Sigma-Aldrich, UK) and cow’s milk protein fractions whey (Lactalis, France) and casein (218680, Sigma-Aldrich, UK) were added at 100µg/mL in all experiments. Cow’s milk allergens BLG (NA-BD5-1, Inbio, UK) and αs-casein (C6780, Sigma-Aldrich, UK) were added at 50µg/mL in all experiments reported. Endotoxin levels of all protein fractions and allergens were assessed by a LAL assay (Charles River, USA) according to manufacturer’s protocol. Cow’s milk protein fractions and allergens contained <1ng endotoxins/mL and OVA 3.6ng endotoxins/mL at the concentration used in the experiments.

### 2.3 Culture of intestinal epithelial cell line HT-29

The human colon adenocarcinoma HT-29 (passages 146-149) was used as an intestinal epithelium model cell line and cultured in 1% penicillin/streptomycin (pen/strep) (Sigma-Aldrich, UK) 10% FCS (Gibco, USA) McCoy’s 5A medium (Gibco, USA). Medium was refreshed every 2–3 days. At 80-90% confluency, cells were washed twice with PBS and trypsinized to passage or seed in transwell plates.

### 2.4 Isolation and culture of moDCs and T cells

PBMCs were isolated from buffy coats from healthy donors (Dutch Blood Bank, Netherlands) by density gradient centrifugation in Leucosep tubes (Greiner, Germany). Monocytes (42-93% purity) and T cells (60-87% purity) were isolated by negative selection according to manufacturer’s protocol (Miltenyi Biotec, Germany). Monocytes were cultured for five days in RPMI 1640 (Lonza, Switzerland) with 10% FCS, 1% pen/strep, 100ng/mL IL4 (Prospec, Israel) and 60ng/mL GM-CSF (Prospec, Israel) to differentiate into immature moDCs. Half of the medium (including cytokines for differentiation) was refreshed every other day. Differentiation was evaluated for donors included in the pHMO experiments and assessed successful with 71-89% CD209+/HLA-DR+ in viable single CD14- cells by flow cytometry (*see 2.7 Flow cytometry*). T cells were stored in 90% FCS and 10% DMSO at -80°C until use in the co-culture model.

### 2.5 (co-)culture models

**Figure 1:**
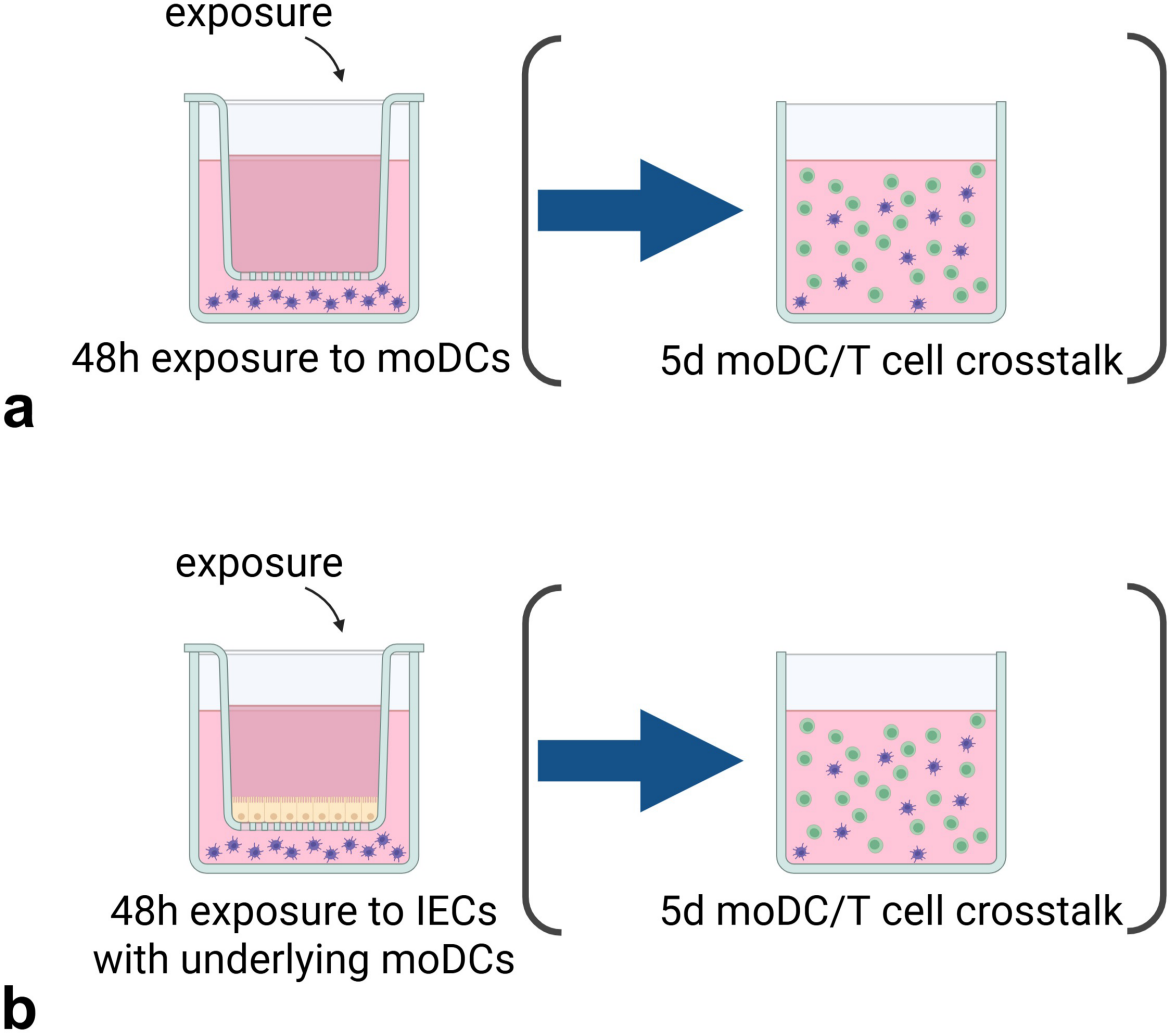
Experimental set-up of the (IEC-)DC exposure and subsequent DC-T cell co-culture. The stimuli are exposed for 48h **(a)** directly to moDCs or added **(b)** apically to HT29 IECs with basolateral moDCs, after which the pre-exposed moDCs are transferred to a 5d DC-T cell co-culture. Created with BioRender.com.

We used co-culture models with and without IECs (see **Figure 1**). For the (IEC-)DC model, HT-29 cells were seeded into 12-well transwell inserts (Corning Costar, US, polyester membrane, 0.4 µm pores) and cultured six days with apical and basolateral medium refreshments every other day including the day before stimulants are added. At day 7, 5x10^5^ moDCs in 1.5mL 10% FCS 1%pen/strep RPMI was added basolaterally in each well. Allergens were added apically in a total volume of 500µl and incubated for 48h. In all experiments, a medium control was included. In the experiments with pHMOs, an LPS control (25ng/mL) (Sigma-Aldrich, UK) was included. In case of a DC only model, allergens were apically added to a transwell insert without IEC seeded on the transwell. After incubation, basolateral supernatant was collected and stored at -20°C until measuring cytokine levels by enzyme-linked immunosorbent assays (ELISA) and phenotype of moDCs was assessed by flow cytometry. HT-29 viability was tested for allergen comparison experiments with an immediate WST assay according to manufacturer’s protocol (Roche, Switzerland) and was not significantly different between conditions (data not shown). For the experiments including pHMOs, HT-29 viability was assessed with Live/Dead Fixable Viability-IF876 (Invitrogen, USA, 1:2000) by flow cytometry and was not significantly different between conditions (data not shown).

For the subsequent DC-T cell model, 100µl with 5x10^4^ pre-exposed moDCs from the (IEC-)DC model was transferred to a 24 well flat-bottom plate. Stored naïve T cells were resuspended in IMDM medium with 5% FCS, 1% pen/strep, 20µg/mL apo-transferrine (Sigma-Aldrich, UK) and 50µM β-mercaptoethanol (Sigma-Aldrich, UK), and added as 5x10^5^ naïve T cells in 400µl medium per well. Pre-exposed moDCs were co-cultured with allogenic naïve T cells in a 1:10 ratio, respectively. A T cell only control with 5x10^5^ naïve T cells was included. Medium was not refreshed during the 5-day incubation. After incubation, supernatant was collected for ELISA measurements, and cells were collected for flow cytometry.

### 2.6 ELISA

Supernatants were collected from the (co-)culture models to measure cytokine secretion. Concentrations of IL-1β, IL4, IL6, IL8, IL10, IL12p70, IL13, IL17A, IL21, IFNγ, TGFβ, TNFα, TSLP (Thermo Fisher Scientific, USA) and IL25, IL33, CCL20, CCL22 (R&D Systems, USA) were assessed according to manufacturer’s protocol. Galectin-9 was measured using an affinity-purified polyclonal antibody and biotinylated affinity-purified polyclonal antibody pair at 0.75µg/mL (R&D Systems, USA), otherwise following the same protocol.

### 2.7 Flow cytometry

MoDCs and T cells collected from the (co-)cultures were transferred to 96-well plates (Costar Corning, USA) and washed with PBS. Cells were stained for viability, after which non-specific binding sites were blocked with human Fc block (BD Biosciences, USA, 1:100) diluted in PBS with 1% bovine serum albumin (Roche, Switzerland) and 2mM EDTA (Sigma-Aldrich, UK). Flow cytometry experiments for allergen comparison experiments were performed on BD FACS Canto II (Becton Dickinson, USA) and viability was assessed with Fixable Viability Dye 780-APC Cyanine 7 (eBioscience, USA, 1:1100). MoDCs were extracellularly stained with titrated volumes of CD14-PerCP Cy5.5 (eBioscience, USA, 1:80), HLA-DR-V450 (BD Biosciences, USA, 1:80), CD209-APC (BD Biosciences, USA, 1:80), CD80-FITC (eBioscience, USA, 1:80), CD86-PE-Cy7 (eBioscience, USA, 1:1280) and OX40L-PE (BD Biosciences, USA, 1:80). Flow cytometry for all other experiments was performed using a CytoFLEX LX (Beckman Coulter, USA) and viability was assessed with Live/Dead Fixable Viability-IF876 (Invitrogen, USA, 1:2000). In this case, the panel was expanded with CD274(PD-L1)-BUV661 (BD Biosciences, USA, 1:80), CRLF2(TSLPR)-APC/Fire750 (Biolegend, USA, 1:200), ST2(IL33R)-AF700 (R&D Systems, USA, 1:120) and CXCR4-PE/Dazzle 594 (Biolegend, USA, 1:80). The T cell phenotype was assessed by extracellular staining with titrated volumes of CD4-PerCP Cy5.5 (Thermo Fisher, 1:320), CD69-PE (Thermo Fisher Scientific, USA, 1:120), CD25-eFluor 450 (eBioscience, USA, 1:100), CD294(CRTH2)-APC (BD Biosciences, USA, 1:100), CD183(CXCR3)-AF488 (BD Biosciences, USA, 1:80), CD154(CD40L)-BV510 (Biolegend, USA, 1:100), CD185(CXCR5)-APC-Cy7 (Biolegend, USA, 1:250), CCR6-BV661 (Biolegend, USA, 1:100), CD194(CCR4)-PE/Dazzle 594 (Biolegend, USA, 1:200), CD161-BV650 (BD Biosciences, USA, 1:100), CD45RO-BUV395 (BD Biosciences, USA, 1:120) and CD45RA-BV605 (BD Biosciences, USA, 1:150). T cells were fixed and permeabilized with the FoxP3/Transcription Factor Staining Buffer Set (eBioscience, USA) and intranuclearly stained with FoxP3-PE-Cy7 (eBioscience, USA, 1:250).

### 2.8 Statistical analysis

Differences between interventions with controls were tested for statistical significance by repeated measures one-way ANOVA followed by Dunnett’s or Sidak’s multiple comparison posthoc test for comparison with only medium control or to medium control and LPS control, respectively. Non-normally distributed data was log- or square root-transformed before testing. In case transformed data did not follow normal distribution, differences were tested with the non-parametric Friedman test with Dunn’s multiple comparison posthoc test. For all tests, a probability <0.05 was considered significant. Data is presented as mean ± SEM for 5–6 independent biological donors per dataset. All analysis were performed with GraphPad Prism 10.

## 3 Results

### 3.1 Isolated pHMOs from Se+ and Se- milk activate moDCs in a dose-dependent manner

MoDCs are essential in the processing and determination of immune responses toward a given antigen, determining T cells responsiveness. Therefore, we first assessed the direct effect of full HMO profile isolates from pooled Se+ and Se- donor milk on moDCs in the DC model. MoDCs were exposed to three physiologically relevant doses of Se+ and Se- pHMOs for 48h (29, 38). Then, pHMO-pre-exposed moDCs were co-cultured with naïve T cells in the DC-T cell model to assess if T cell phenotype was affected.

**Figure 2:**
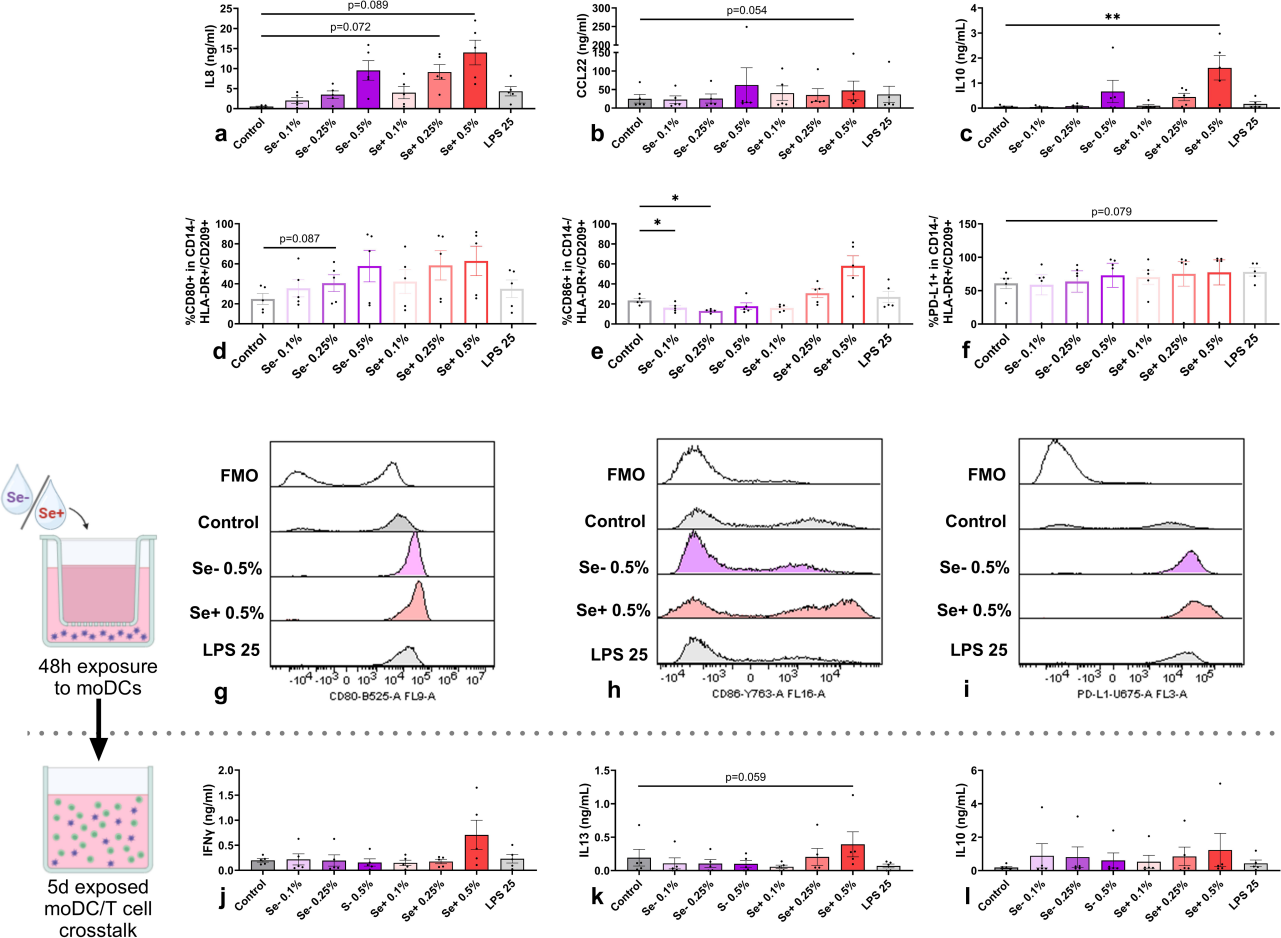
Effects of Se- and Se+ pHMOs on moDCs (upper panel) and subsequent effect of pHMO-pre-exposed moDCs on naïve T cells after co-culture (lower panel). MoDCs are exposed to Se- and Se+ pHMOs for 48h after which **(a)** IL8, **(b)** CCL22 and **(c)** IL10 concentration in basolateral supernatant was measured. In addition, the percentage of CD14-/HLA-DR+/CD209+ moDCs expressing surface markers **(d)** CD80, **(e)** CD86 and **(f)** PD-L1 was assessed, presented with representative histograms (g for CD80, h for CD86 and i for PD-L1), with respective fluorescence minus one control (FMOs). After a 5-day co-culture of pHMO-pre-exposed moDCs with naïve T cells, supernatant levels of **(j)** IFNγ, **(k)** IL13 and **(l)** IL10 were measured. Data is presented as mean ± SEM. Differences between interventions with medium control, and Se+ 0.5% with LPS 25 (ng/mL) control, were tested by repeated measures one-way ANOVA with Sidak’s multiple comparison posthoc test or Friedman with Dunn’s multiple comparison posthoc test. *p ≤ 0.05, **p ≤0.01. n=5 independent biological replicates, each data point represents the value of an individual donor **(a-i)** or an independent allogenic donor pair (DC with T cell) **(j-l)**. Data shown are pooled from two independent experiments. Schematic experimental designs created with BioRender.com.

Although without reaching statistical significance, Se+ and Se- pHMOs seemed to activate moDCs in a dose-dependent manner as shown by the increased secretion of IL8 (**Figure 2a**) and %CD80+ moDCs (**Figure 2d**) with increasing pHMO concentration. Only Se+ pHMOs tended to skew moDCs toward a type 2-associated phenotype as assessed by an increased tendency of CCL22 secretion (p=0.054, 0.5% compared to control) (**Figure 2b**) and a similar pattern for CD86 expression in representative samples (**Figure 2h**) and %CD86+ moDCs (**Figure 2e**). By contrast, both 0.1% and 0.25% Se- pHMOs decreased the %CD86+ moDCs. Se+ 0.5% pHMOs significantly increased IL10 concentration (**Figure 2c**). Likewise, Se+ pHMOs seemed to induce inhibitory marker PD-L1 expression in representative samples (**Figure 2i**) and %moDCs expressing inhibitory marker PD-L1 (p=0.079 for 0.5%) (**Figure 2f**). Of note, one donor had a relatively low baseline %PD-L1+ moDCs, and only in this donor the %PD-L1+ moDCs did not increase in response to the pHMOs. The highest dose of Se+ and Se- pHMOs did not affect moDC viability after 48h exposure, although the highest dose of Se+ pHMOs tended to increase cell viability compared to the LPS control (p=0.086) (**Supplementary Figure S1**). In sum, these data indicate that even though donor variations are high, Se+ and Se- pHMOs both tend to activate moDCs in a dose-dependent manner with Se+ pHMOs inducing the most potent effect, leading toward a regulatory and more type 2-associated phenotype of moDCs.

As moDCs directly interact with T cells, we studied subsequent effects of pHMOs on T cell responses by co-culturing pHMO-pre-exposed moDCs with naïve T cells. After five days, cytokine concentration in supernatants were compared with the controls. Although IFNγ secretion by T cells co-cultured with 0.5% Se+ pHMO-pre-exposed moDCs showed an inclining pattern, this did not reach significance (**Figure 2j**). Likewise, IL13 secretion tended to be increased for the 0.5% Se+ pHMO condition, compared to the control (p=0.059) (**Figure 2k**). While the concentration of IL10 in the supernatant of moDCs exposed to 0.5% Se+ pHMOs was increased (**Figure 2c**), there was no effect of the pHMOs-pre-exposed moDCs on the T cell response observed after the 5-day co-culture (**Figure 2l**). Thus, although Se+ and Se- pHMOs activated moDCs, pHMO-pre-exposed moDCs did not significantly impact overall cytokine secretion by T cells in the absence of any allergenic trigger.

Considering the low levels of endotoxins that remain present in the isolated pHMOs after endotoxin removal, we included an LPS control equal to the endotoxin level for the condition with highest level of remaining endotoxins; 0.5% Se+ pHMOs. Importantly, the low dose (25ng/mL) LPS control did not significantly increase cytokine release or co-stimulatory molecule expression by moDCs compared to the medium control or 0.5% Se+ pHMOs (**Figures 2a–f**). Similarly, we did not observe differences of cytokine secretion after the exposed DC-T cell co-culture compared to the medium control or 0.5% Se+ pHMOs (**Figures 2j–l**).

### 3.2 Cow’s milk allergen BLG and protein fraction casein enhance type 2 CCL22 release by moDCs

To study the immunomodulatory effect of pHMOs in the context of food allergen exposure, we first determined the type 2 immune activating capacity of cow’s milk proteins and isolated allergens within the (IEC-)DC model, capable of distinguishing between allergenic and non-allergenic proteins (39). Hen’s egg allergen OVA was used as a positive control as in previous studies we showed its type 2-driving effects in a similar model (34).

**Figure 3:**
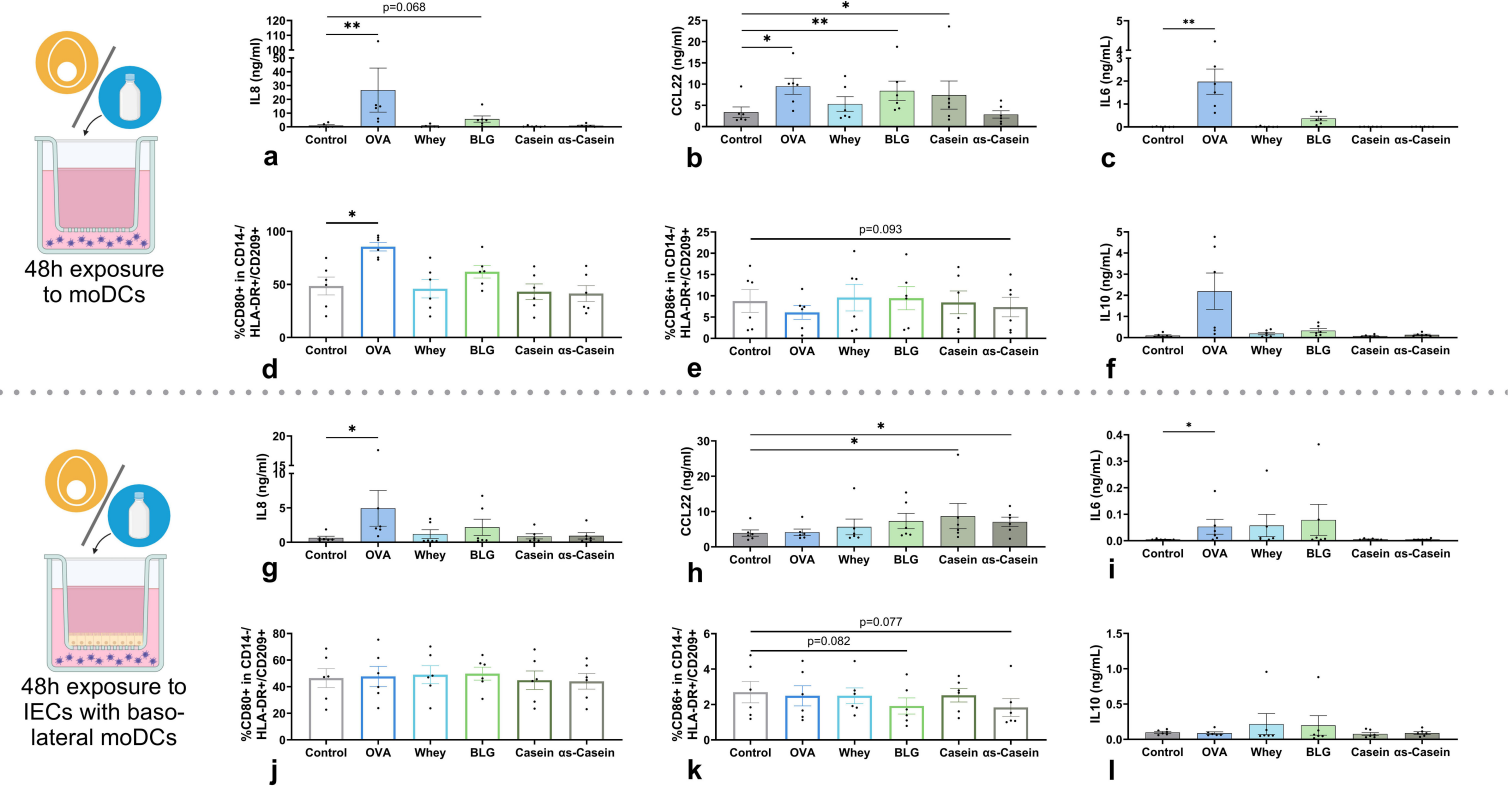
Effects of cow’s milk protein fractions and isolated allergens on moDCs, in the absence (upper panel) or presence of IECs (lower panel). MoDCs were exposed to 45 for 48h in the absence/presence of IECs HT-29 after which **(a, g)** IL8, **(b, h)** CCL22, **(c, i)** IL6 and **(f, l)** IL10 concentration in basolateral supernatant was measured. In addition, the percentage of CD14-/HLA-DR+/CD209+ moDCs expressing surface markers **(d, j)** CD80 and **(e, k)** CD86 was measured. OVA is included as a positive control for immune modulation. Data is presented as mean ± SEM. Differences between interventions with medium control were tested by repeated measures one-way ANOVA with Dunnett’s multiple comparison posthoc test or Friedman with Dunn’s multiple comparison posthoc test. *p ≤ 0.05, **p ≤0.01. n=6 independent biological replicates, each data point represents the value of an individual donor. Data shown are pooled from two independent experiments. Schematic experimental designs created with BioRender.com.

In the absence of IECs, exposure to allergens OVA, and to a lesser extent BLG, activated moDCs as shown by the increased IL8 concentration (**Figure 3a**) and increased %CD80+ moDCs (**Figure 3d**) compared to the control. In addition, OVA, BLG and casein increased the CCL22 secretion (**Figure 3b**) while the %CD86+ moDCs was not increased (**Figure 3e**). None of the allergens affected %OX40L+ moDCs (**Supplementary Figure S2**). Of all allergens, only OVA increased IL6 secretion (**Figure 3c**) and none of the allergens significantly affected secretion of the regulatory cytokines IL10 (**Figure 3f**) and TGFβ (**Supplementary Figure S2**). The concentration of alarmins IL33, IL25 and TSLP, pro-inflammatory cytokine IL1β, type 1-associated IL12p70 and chemokine CCL20 were below detection level (data not shown).

When allergens were added apically to IECs with basolateral moDCs, the effects described above were mostly dampened. However, OVA exposure still significantly increased IL8 secretion (**Figure 3g**), while %CD80+ moDCs was not affected (**Figure 3j**). In addition, only casein and αs-casein significantly enhanced CCL22 secretion (**Figure 3h**). However, BLG and αs-casein tended to decrease %CD86+ moDCs compared to control (**Figure 3k**). None of the allergens affected %OX40L+ moDCs (**Supplementary Figure S2**). As observed without IECs, only OVA induced IL8 and IL6 secretion (**Figures 3g, i**) and none of the allergens altered secretion of the regulatory cytokines IL10 (**Figure 3l**) or TGFβ (**Supplementary Figure S2**).

Together, all allergens were able to modulate the moDCs in the absence of IECs, with BLG inducing the most specific type 2-associated response. IECs dampened most of the effects but OVA had the strongest moDC-activating effect, partly sustained in the presence of IECs.

### 3.3 In the presence of BLG, pHMOs from Se+ and Se- milk induce tolerance-associated immune markers

The impact of pHMOs on allergen-induced mucosal immune responses was studied by co-exposing moDCs to BLG, the main allergen in whey, with Se+ or Se- pHMOs in the DC model. We included cow’s milk allergen BLG in our experiments as cow’s milk allergy presents a major burden during early life (2, 4).

**Figure 4:**
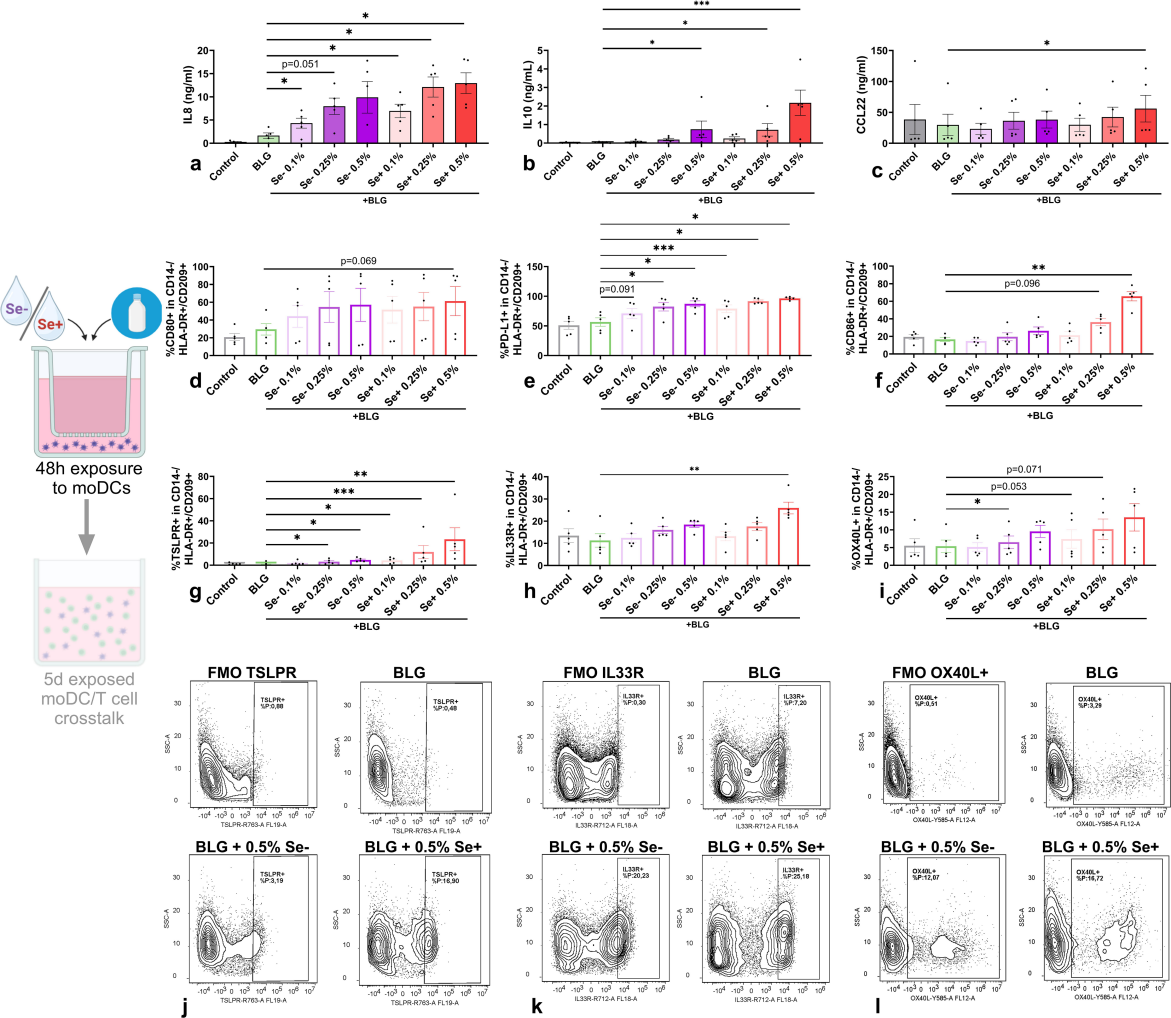
Effects of Se- and Se+ pHMOs with BLG on moDCs. MoDCs were exposed to BLG and pHMOs for 48h after which **(a)** IL8, **(b)** IL10 and **(c)** CCL22 concentration in basolateral supernatant was measured. In addition, the percentage of CD14-/HLA-DR+/CD209+ moDCs expressing surface markers **(d)** CD80, **(e)** PD-L1 and **(f)** CD86, **(j)** TSPLR, **(k)** IL33R and **(l)** OX40L were assessed. A representative sample of CD14-/HLA-DR+/CD209+ moDCs expressing **(g)** TSLPR, **(h)** IL33R and **(i)** OX40L is presented with respective fluorescence minus one control (FMOs). Data is presented as mean ± SEM. Measurements for medium control and interventions were compared with BLG by repeated measures one-way ANOVA with Dunnet’s posthoc test or Friedman test. *p ≤ 0.05, **p ≤0.01, ***p ≤ 0.001. n=5 independent biological replicates, each data point represents the value of an individual donor. Data shown are pooled from two independent experiments. Schematic experimental design created with BioRender.com.

In the presence of BLG, not only Se+ pHMOs but also Se- pHMOs exposed moDCs increased IL8 (**Figure 4a**) and CCL20 (**Supplementary Figure S1**) secretion compared to exposure to BLG only. 0.5% Se+ pHMOs tended to increase the %CD80+ moDCs (p=0.069), and a similar pattern was observed for Se- pHMOs (**Figure 4d**). The %CD80+ moDCs was consistently increased for all five donors after exposure to 0.5% Se+ pHMOs compared to BLG only, however, the variation in effect size contributed to the broad variation in response. Se+ pHMOs induced a more pronounced type 2-associated response, based on the increased %CD86+ (**Figure 4f**) and %IL33R+ (**Figures 4h, k**) moDCs with %OX40L+ showing the same tendency (**Figures 4i, l**). Similarly, exposure to BLG and 0.5% Se+ pHMOs also significantly increased CCL22 secretion, although inter-donor variation was observed (**Figure 4c**). BLG with Se- pHMOs also increased the %TSLPR+ (**Figures 4g, j**) and %OX40L+ (**Figures 4i, l**) moDCs compared to exposure to BLG only. Profoundly, both Se+ and Se- pHMOs increased the secretion of the regulatory cytokine IL10 (**Figure 4b**) and the %moDCs expressing inhibitory markers PD-L1 (**Figure 4e**). These data indicate that Se+ and Se- pHMOs influenced the moDC response in the presence of cow’s milk allergen BLG, with distinct effects on moDC activation and inducing a type 2 as well as a regulatory phenotype.

**Figure 5:**
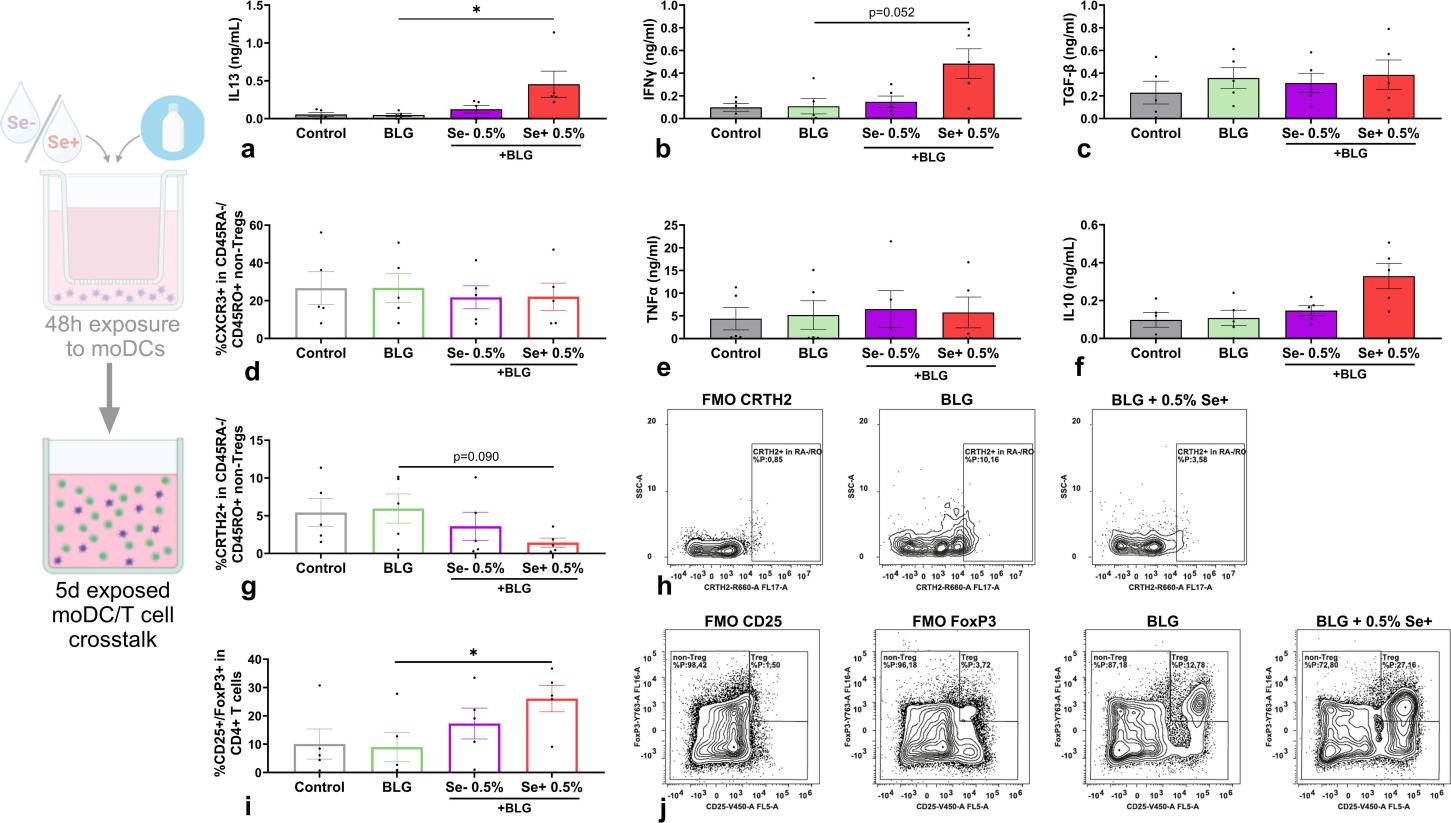
Effects of moDCs pre-exposed to Se- or Se+ pHMOs with BLG on T cells. Naïve T cells were co-cultured for five days with BLG- and/or pHMO-pre-exposed moDCs after which **(a)** IL13, **(b)** IFNγ, **(c)** TGFβ, **(e)** TNFα and **(f)** IL10 concentration in supernatant was measured. In addition, the percentage of CD4+/CD25-/FoxP3-CD45RA-/CD45RO+ T cells expressing **(d)** CXCR3 and **(g)** CRTH2 and **(i)** CD4+ cells expressing CD25+/FoxP3+ was assessed. A representative sample of **(h)** CD4+/CD25-/FoxP3-/CD45RA-/CD45RO+ T cells expressing CRTH2 and **(j)** CD4+ expressing CD25+/FoxP3+ is presented with respective fluorescence minus one control (FMOs). Data is presented as mean ± SEM. Measurements for medium control and interventions were compared with BLG by repeated measure one-way ANOVA with Dunnet’s multiple comparison posthoc test or Friedman test with Dunns multiple comparison posthoc test. *p ≤ 0.05. n=5 independent biological replicates, each data point represents the value of an independent allogenic donor pair (DC with T cell). Data shown are pooled from two independent experiments. Schematic experimental design created with BioRender.com.

To evaluate the effect of moDCs exposed to BLG and pHMOs on T cell polarization, pre-exposed moDCs were co-cultured with naïve T cells. After a 5-day co-culture, cytokine secretion was measured in supernatants (**Figures 5a–c, e, f**) and T cell phenotype was assessed by flow cytometry (**Figures 5d, g–j**). T cells, co-cultured with moDCs pre-exposed to BLG with 0.5% Se+ pHMOs, showed increased IL13 secretion (**Figure 5a**) compared to BLG only, although the percentage of memory T cells (CD4+/CD45RO+/CD45RA-) expressing Th2 marker CRTH2 was decreased (**Figures 5g, h**). At the same time, IFNγ concentration after co-culture with moDCs pe-exposed to BLG and 0.5% Se+ pHMOs tended to increase (p=0.052) (**Figure 5b**), compared to only BLG, without affecting type 1-associated inflammatory markers TNFα (**Figure 5e**) and %CXCR3+ T cells (**Figure 5d**). The percentage of Tregs increased (**Figures 5i, j**) after co-culture of T cells with moDCs pre-exposed to BLG and Se+ pHMOs. The IL21 and galectin-9 concentration remained unaffected, whereas concentrations of IL4 and IL17A were below the detection limit (**Supplementary Figure S4**).

Hence, in the presence of cow’s milk allergen BLG, Se+ pHMO-pre-exposed moDC increased the percentage of Tregs as well as supported type 2 cytokine release by T cells. Se- pHMO-pre-exposed moDCs did not show this effect on T cell polarization.

### 3.4 Co-exposure of BLG with Se- or Se+ pHMOs via IECs reduces %CD86+ moDCs

This differential effect of pHMOs on moDCs was subsequently tested in the presence of IECs in the IEC-DC model. BLG and pHMOs were added apically to IECs with basolateral moDCs.

**Figure 6:**
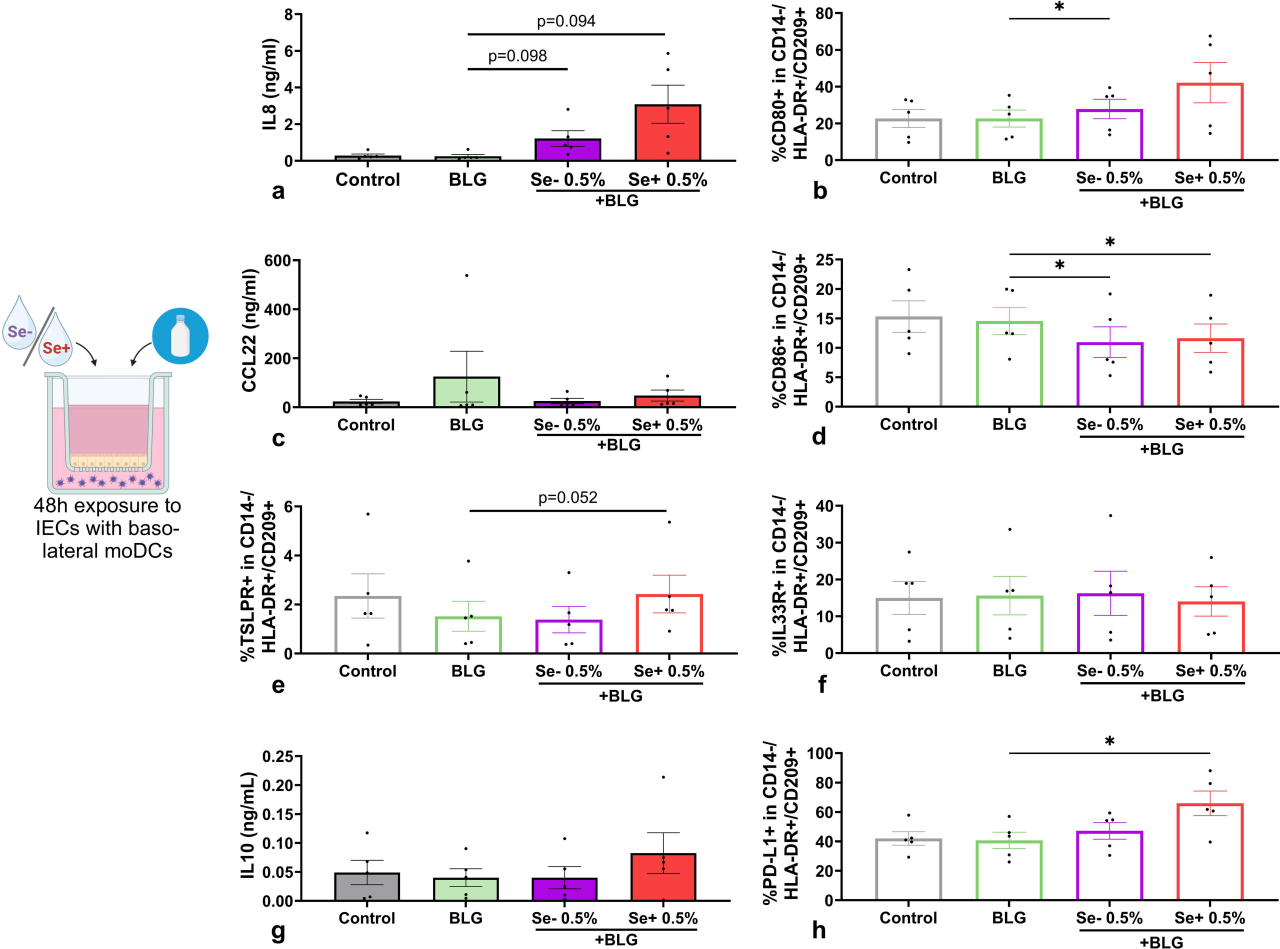
Effects of non-secretor (Se-) or secretor (Se+) pHMOs with BLG on moDCs via IECs. IECs HT-29, with underlying moDCs, were apically exposed to BLG and pHMOs for 48h after which **(a)** IL8, **(c)** CCL22, **(g)** IL10 concentration in basolateral supernatant was measured. In addition, percentage of CD14-/HLA-DR+/CD209+ moDCs expressing surface markers **(b)** CD80, **(d)** CD86, **(e)** TSLPR, **(f)** IL33 and **(h)** PD-L1 was measured. Data is presented as mean ± SEM. Measurements for medium control and interventions were compared with BLG by repeated measures one-way ANOVA with Dunnet’s posthoc test or Friedman with Dunn’s posthoc test. *p ≤ 0.05. n=5 independent biological replicates, each data point represents the value of an individual donor. Data shown are pooled from two independent experiments. Schematic experimental design created with BioRender.com.

Also in the presence of BLG-exposed IECs, pHMOs tended to increase IL8 concentration (**Figure 6a**) in a IEC-DC co-culture, and CCL20 was significantly increased compared to BLG only (**Supplementary Figure S1**). When added to IEC in presence of BLG, Se- pHMOs increased %CD80+ moDCs basolaterally, compared to BLG alone, and Se+ pHMOs followed the same pattern (**Figure 6b**), while type 2 CCL22 secretion was not affected (**Figure 6c**). When exposed to IEC combined with BLG, both Se+ and Se- pHMOs significantly reduced %CD86+ moDCs basolaterally compared to BLG only (**Figure 6d**). The basolateral %TSLPR+ (**Figure 6e**) moDCs tended to increase in 0.5% Se+ pHMO IEC-exposed conditions whereas the %IL33R moDCs remained unaffected (**Figure 6f**). The %PD-L1+ moDCs increased upon IEC-DC exposure to 0.5% Se+ pHMOs with BLG (**Figure 6h**), but regulatory IL10 concentrations (**Figure 6g**) or galectin-9 (**Supplementary Figure S1**) were not affected.

The activation status of moDCs derived from pHMO- and BLG-exposed IEC-DC co-cultures was less pronounced, when compared to directly exposed moDCs. When these IEC-DC derived moDCs were co-cultured with naïve T cells, the T cell responses silenced both for Se+ as well as Se- pHMOs with BLG conditions (**Supplementary Figures S3**, **S4**).

## 4 Discussion

**Figure 7:**
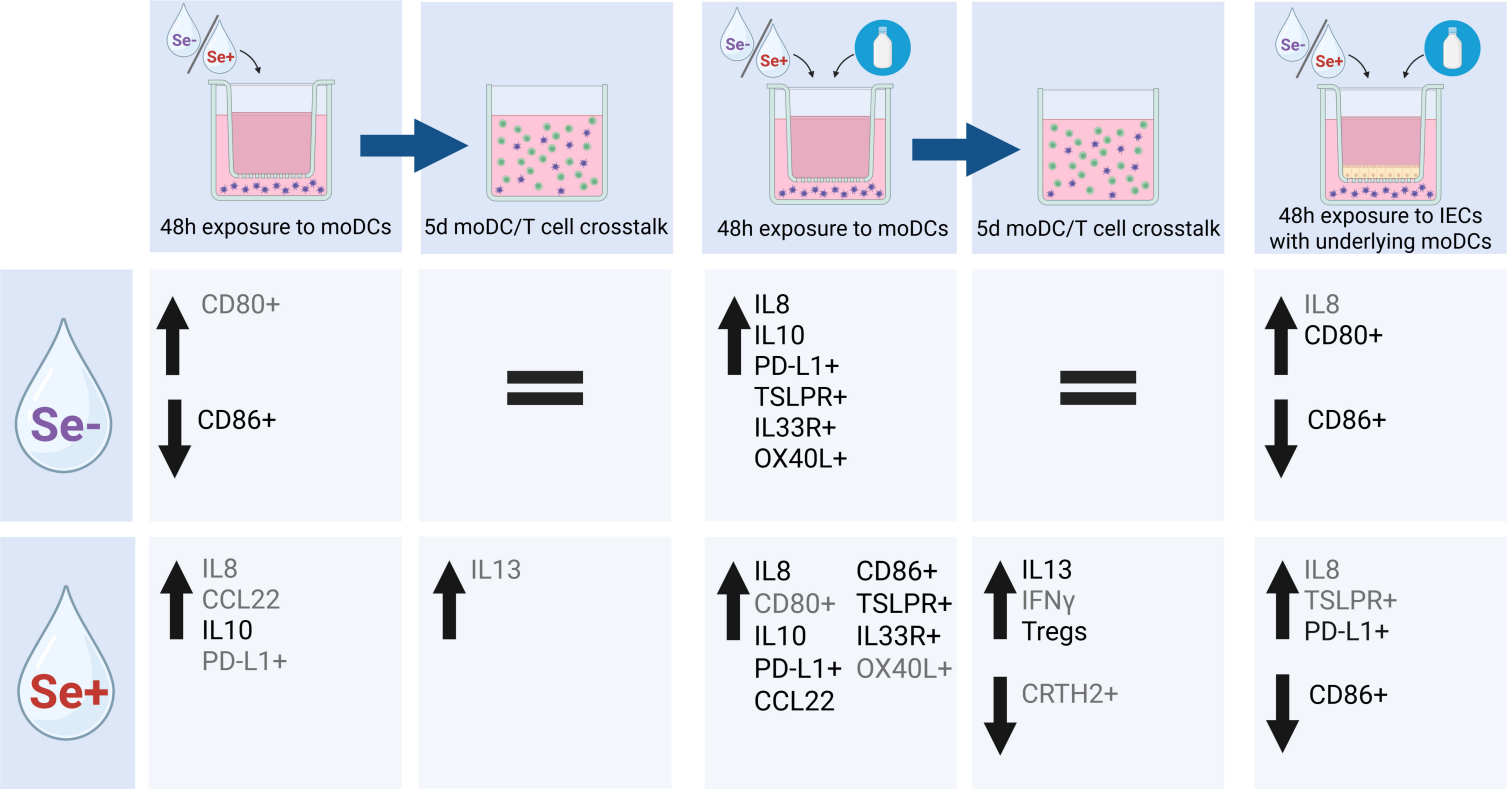
Schematic overview of effect of Se- and Se+ pHMOs with and without BLG on DC or via IEC-DC and T cell response of subsequent DC-T cell co-culture. Significant (p<0.05) differences in cytokine concentration or marker expression are depicted in black, while trends (p<0.1) are depicted in grey. Created with BioRender.com.

Early life exposure to food allergens via the gastrointestinal mucosa is of pivotal importance for the development of oral tolerance (40, 41). The gut microbiome drives early life maturation of the mucosal immune system and HMOs in human milk are known to support microbiome development (42). Specific HMOs, however, may also have direct effects on mucosal immune development by acting on intestinal epithelial cells and/or dendritic cells that can drive either tolerance or instruct immunity to food derived proteins (28, 34). Understanding the impact of environmental and possible protective factors, including human milk components such as HMOs, when exposing infants to food allergens is crucial to reduce the high burden of food allergies. Breastfeeding is the optimal nutrition for infants and contains a wide range of bioactive compounds benefiting healthy development of the immune system. However, to our knowledge, the effect of the full profile of isolated HMOs from Se+ or Se- milk in the context of food allergen sensitization has not been studied before. We found that both HMO profiles activate moDCs in a dose-dependent manner. In the presence of cow’s milk allergen BLG, only Se+ pHMOs increased type 2-associated markers CCL22 secretion and the %CD86+ and %IL33R+ moDCs, while both Se+ and Se- increased regulatory IL10 secretion and inhibitory %PD-L1+ moDCs. IECs dampened the immunomodulatory effect of BLG and/or pHMOs. Even though Se+ and Se- pHMOs, when exposed to IEC-DC co-cultures with BLG, still tended to increase some moDC markers, these pHMO- and BLG-pre-exposed moDCs did not induce T cell activation. Together, these data suggest that pHMOs differentially modulate the immune response in our models dependent on the secretor status (see **Figure 7** for overview).

Since clear differences in the HMO composition are naturally present, we included Se+ and Se-pHMOs in our model, rather than a combined sample. Se+ milk has generally higher levels of total HMOs, higher levels of fucosylated HMOs and fucose bound via an α1,2-linkage (43, 44). More specifically, Se+ milk is characterized by high levels of 2’-FL, LNFP I and LNDFH I as opposed to milk from Se- mothers (23, 43, 45). In contrast, Se- milk generally has lower levels of fucosylated HMOs and 3-FL and lacto-*N*-fucopentaose III (LNFP III) are relatively more abundant. Dependent on FUT3 gene activity, also lacto-*N*-fucopentaose II (LNFP II) and lacto-*N*-difucohexaose II (LNDFH II) are present in Se- milk (23, 43). Indeed, our Se+ pHMO samples contained high levels of 2’-FL, also compared to our Se- pHMO sample. In contrast, Se- pHMO samples contained relatively high levels of LNT, namely more than twice as abundant compared to Se+ pHMO samples.

These HMO-compositional differences might translate to differential effects on immune cells. Different lectin receptors expressed by immune cells, including monocytes and DCs, interact with HMOs and are involved in immunomodulatory pathways. To illustrate, C-type lectin receptor DC-specific ICAM-grabbing non-integrin (DC-SIGN) specifically binds fucosylated HMOs, as nicely reviewed by Triantis and colleagues (46). We observed several consistent differences between Se+ and Se- pHMOs on immune cells. Se+ pHMOs seems to induce a stronger activating effect with a tendency of increased IL8 secretion. The immune response seems to be type 2-associated, as indicated by a tendency to increased CCL22 secretion and inclining pattern of %CD86+ moDCs, and tendency of increased IL13 secretion after co-culture with naïve T cells. One of the hallmark Se+ HMOs is 2’-FL. Several studies investigated the immunomodulatory effect of specifically 2’-FL on non-activated moDCs but generally found little to no effect on assessed markers (34, 47, 48). In our current DC model, in contrast to Se+ pHMOs, Se- pHMOs did not tend to induce IL8 or CCL22 secretion by moDCs, but tended to increase %CD80 moDCs. Xiao and colleagues previously showed that pHMOs (without distinguishing secretors and non-secretors) induced tolerogenic characteristics in immature moDCs after exposure, with increased IL10, IL27 and IL6 secretion and unaffected pro-inflammatory IL12p70 and TNFα secretion by exposed moDCs, which also induced IL10 secretion after co-culture with naïve T cells (33). We showed that Se+ pHMOs also increased IL10 secretion and tended to increase the %PD-L1+ moDCs, while Se- pHMOs reduced co-stimulatory molecule %CD86+ moDCs, all features of the semi-mature tolerogenic DCs (tDCs) (33, 49, 50). Although, this did not directly translate to the T cell response as the IL10 secretion was unaffected. Instead, Se+ pHMO-pre-exposed moDCs supported T cells which tended to secrete IL13, while T-cells cultured with Se- pHMO-pre-exposed moDCs remained silenced. The tolerogenic effect of pHMOs in the study by Xiao et al. was especially shown in the context of an inflammatory trigger. In the presence of LPS, pHMOs reduced the expression of co-stimulatory markers compared to LPS alone, whereas in absence of LPS, pHMOs increased the expression of these markers (33).

Differences in HMO-induced responses between studies might be explained by the presence or absence of an additional environmental trigger. As described above, the presence of high LPS levels can make the difference in the expression of co-stimulatory molecules (33). Perdijk et al. demonstrated that 3’-SL induced semi-mature tDCs with reduced %CD86+ moDCs, increased IL10 and reduced IL12p70 and TNFα secretion. However, LPS removal from 3’-SL diminished these effects (51). This advocates for consideration of LPS levels in HMO samples exposed to immune cells in *in vitro* studies to distinguish HMO and LPS effects. On the other hand, in the *in vivo* situation, HMOs interact with DCs in an environment with microbiota including LPS and other microbial components (52). In our study, Se+ and Se- pHMO samples contained low levels of endotoxin after LPS removal, which may have interfered in the experiment. We therefore included a LPS control for the pHMO condition with the highest LPS levels as present in 0.5% Se+ pHMOs (25ng/mL). The LPS control did not significantly affect any of the assessed markers, although the increased %PD-L1+ moDCs after exposure to Se+ pHMOs might be partly to LPS contamination. Therefore, we concluded that interference of endotoxins was limited in our study.

We were interested in the differential effect of Se+ and Se- pHMOs in the context of a sensitizing trigger, cow’s milk allergen BLG. Previously, the preventative effect of 2’-FL and 3-FL on OVA-activated IECs on moDCs and subsequent naïve T cell response was studied (34). OVA activated IECs despite the pre-incubation with 2’-FL and 3-FL. Subsequently, 2’-FL and OVA pre-exposed IECs co-cultured with moDCs, induced both a type 2 and 1 inflammatory as well as regulatory response when the collected moDCs were cultured with T cells, while 3-FL and OVA primed IEC-exposed moDCs inhibited IL13 secretion by T cells compared to OVA exposure alone, in this sequential IEC-DC and DC-T cell allogenic co-culture models. In absence of IECs, we observed that both Se+ and Se- pHMOs with simultaneous BLG exposure activated moDCs with increased IL8 secretion and %CD86+, with combined Se+ pHMOs and BLG also inducing CCL22 secretion and %TSLPR+ moDCs, compared to BLG only. Co-culture of Se+ pHMO- and BLG-pre-exposed moDCs with T cells resulted in increased IL13 as well as a tendency toward increased IFNγ secretion, while Se- pHMOs did not affect cytokine secretion compared to BLG only. Interestingly, if we exposed moDCs to the pHMOs with BLG applied via IECs, in these IEC-DC co-cultures, the %CD86+ moDCs was reduced, indicating reduced maturity. Specifically Se+ pHMOs with BLG increased inhibitory receptor %PD-L1+ moDCs from IEC-DC co-cultures, and these moDCs could not activate T cells. A study exposing moDCs to OVA in the presence or absence of IECs also observed that OVA-pre-exposed moDCs directed toward a type 2-associated response (53). However, IECs prevented Th2 polarization and inhibited moDC maturation. When IECs were pre-exposed to OVA prior to co-culture with moDCs, these pre-exposed IEC were activated but incapable of inducing moDCs maturation during the subsequent co-culture (34). OVA was removed prior to IEC-DC co-culture, therefore preventing allergen uptake in moDCs. Since antigen uptake is essential for moDC maturation, this could explain the lack of maturation seen in this IEC-pre-exposure model (54). In our IEC-DC model, IECs might limit direct contact between basolateral moDCs and apical stimuli, therefore dampening the pHMO- and BLG-activating effect on moDCs and resulting in immature moDCs, limiting the capacity of driving T cell activation.

Generally, HMOs are thought to skew the immune response toward a type 1 and regulatory response (28). We show that this may apply for Se+ pHMOs in presence of BLG, but beyond a regulatory type of immune response, also a type 2 response was observed. Markers for DCs skewing the T cell phenotype toward Th2 are not as clearly defined as, for example, Th2 cells. Even though CCL22, CCL17, and surface CD86 and OX40L expression are associated with type 2 skewing, it is important to study the functional outcome of moDC priming by studying their function in naïve T cell instruction. Type 2-associated DCs are therefore primarily identified by their ability to drive Th2 polarization (55, 56). Our data indicate that moDC exposure to pHMOs induced markers associated with a tDC phenotype. Interestingly, moDCs exposed to Se+ pHMOs and BLG drive T cells toward a type 2 as well as regulatory T cell response. Indeed, the helminth-associated glycan LNFP III, also present in Se+ and Se- milk, was shown to induce IL4 and reduce IFNγ secretion by OVA-specific CD4+ T cells after co-culture with HMO-exposed moDCs indicating DC2 maturation (57). We found that the effects of Se+ pHMOs with BLG on moDCs were to some extent translated into the T cell response as IL13 secretion increased, although the %CRTH2+ memory Th2 cells (CD4+/CD45RO+/CD45RA- non-Treg) tended to decrease. Interestingly, Se+ pHMO-pre-exposed moDCs significantly increased the percentage of regulatory T cells (CD4+/CD25+/FoxP3+). Together, it seems that individual or pooled HMOs may have different effects on the immune response, dependent on the specific HMO or composition, and the context in which an allergen is presented.

Here we studied the effect of pHMOs in the presence of an allergenic trigger. For this, we compared the effects of cow’s milk proteins relevant during early life on moDCs. We included whole cow’s milk protein whey and casein and the main allergens in these factions, BLG and αs-casein, respectively. Hen’s egg allergen OVA was used as a positive control as it was previously shown to have sensitizing capacity in different variations of the model (34, 53, 58). Cow’s milk allergy is more common during early life and presents a major burden with most infant formulas being based on cow’s milk (4). Although OVA induced a stronger activating effect on moDCs than BLG, BLG had a more selective type 2-associated response. It might be that the immunomodulatory effect of pHMOs is dependent on the strength of the environmental trigger to which moDCs are exposed to. As described above, pHMOs themselves induced expression of co-stimulatory molecules by moDCs. Another study observed that, when co-exposed with LPS as an environmental trigger, pHMOs reduced the expression of several co-stimulatory molecules by moDCs compared to the LPS control (33). This was also observed for 0.01% and 0.05% 2’-FL, 3-FL, 3’-siallyllactose (SL), 6’-SL or lacto-*N*-neotetraose (LNnT) exposed to PBMCs. Exposure to non-activated PBMCs led to little to no effect on secreted cytokine profile, while addition to αCD3/CD28-activated PBMCs showed differential regulatory and type 1 skewing effects (59). Similar effects were shown for exposure of 2’-FL on IECs with underlying non-activated PBMCs or αCD3/CD28-activated PBMCs (48). A stronger activating signal, such as OVA, may enhance the regulatory modulatory impact of HMOs, while a more subtle trigger, like BLG, may not be sufficient in type 2 activation, allowing HMOs to effectively modulate the immune response toward a more regulatory and type 1-skewed response. A stronger activating signal might magnify differences between the Se+ and Se- HMO profiles *in vitro*. This may provide future opportunities to include sequential mucosal food allergen sensitization model also implementing steps beyond T cell polarization (53).

The presented types of *in vitro* models help to explore whether HMOs have direct immunomodulatory effects in presence or absence of mucosal triggers, such as food allergens. Future research should further explore potential differences in the immunomodulatory effects on allergic sensitization between Se+ and Se- HMOs by considering increasing allergen levels, as indicated above, or selecting an allergen with a more robust type 2 response. In addition, we added the Se+ and Se- pHMOs in equal dosages to the model. In the real-life situation, Se- milk generally contains a lower level of total HMOs than Se+ milk (60). For example, in the Canadian CHILD cohort, the nineteen most abundant HMOs were quantified, considered to account for over 90% of total HMO content. Se- milk in the CHILD cohort contained 8.94 ± 1.51µmol/mL, whereas Se+ milk contained 15.91 ± 2.80µmol/mL of these nineteen most abundant HMOs (43). Direct translation from our *in vitro* models to the *in vivo* early life situation is also limited because moDCs and T cells in our models were derived from PBMCs of an adult donor and may overestimate local neonatal immune response. Neonate’s immune system is less matured and therefore may respond more subtle but similarly balanced (61). More specifically, IFNγ as well as IL4 and IL5 secretion is reduced by stimulated blood-derived mononuclear cells from infants compared to their parents, although the ratio between type 1 to type 2 cytokines is similar. For the secretion of other cytokines, such as IL10 and IL13, there is not a clear difference between infants and their parents. In the *in vivo* situation, most HMOs would be exposed to the local mucosal immune system, while part of the HMOs crosses the IEC barrier and enter the circulation (29, 31). Similarly, we used the carcinogenic adult-derived non-polarized HT-29 cell line to model IECs in our IEC-DC co-culture model, since these cells previously were found more responsive to allergen exposure compared to the carcinogenic adult-derived polarized Caco-2 cells (39). However, isolated and cultured intestinal enteroids from children and adults revealed differences, showing that pediatric epithelial cell height as well as resistance was reduced compared to adult-derived enteroids (62). More importantly, secretion of cytokines such as IL8, IFNγ and IL6 was absent or limited (below detection level) by pediatric enteroids, while adult-derived enteroids did produce detectable quantities. This suggests that pediatric enteroids may respond differently from than adult-derived cells. On the other hand, in a recent study, immunomodulation through HT-29 was compared to a non-carcinogenic fetal IEC cell line using a IEC-PBMC co-culture (63). This resulted in a similar type 1-associated and regulatory response after activation and exposure to bacterial trigger CpG. Hence, the primary fetal IEC cell line showed a similar but even more sensitive immunomodulatory effect compared to HT-29 cells, as indicated by induced type 1- and type 2-associated as well as regulatory cytokine induction, after additional exposure to non-digestible oligosaccharides. These studies indicate that HT-29 and primary human fetal intestinal epithelial cells can respond similarly in spite of their difference in origin. However, to further study the role of intestinal epithelial cells in mucosal immunomodulatory effects of HMOs, future studies could make use of primary IEC or pediatric cell line models. The availability of clinical studies with human milk are limited due to ethical reasons. Therefore, studies with HMO-supplemented infant formula are performed as Schönknecht et al. recently reviewed (64). Two randomized trials compared cow’s milk allergic symptoms between infants who received whey-based extensively hydrolyzed formula with 2’-FL and/or LNnT to infants receiving similar commercially available formula without HMOs as a secondary outcome, with no significant differences between the groups (65, 66). As supplementing full HMO profiles would be a major undertaking, these effects could be studied in animal models as a more feasible alternative, which would allow us to study the effects after prolonged exposure. This might also provide opportunities to include the role of secretor status of the HMO-receiving offspring in the research.

This *in vitro* study provided a first comparison of a full HMO profile from Se+ and Se- milk on the immune response in the context of allergen exposure. Exposure to Se+ or Se- pHMOs both activated moDCs, regardless of the presence of cow’s milk allergen BLG. Se+ pHMOs activated in a slightly more type 2-associated response if Se+ pHMOs were co-exposed with BLG. Se+ and Se- pHMOs both induced some regulatory features in moDCs which was especially clear for Se+ pHMOs in the presence of BLG, also leading to an increased regulatory T cell response, albeit also a type 2 signature developed. These data show that HMOs from secretor and non-secretor milk differentially modulate the immune response *in vitro*. This highlights the need to further study the relevance of these differences and how they influence the risk of developing food allergies and other allergic diseases for breastfed infants.

## Data Availability

The raw data supporting the conclusions of this article will be made available by the authors, without undue reservation.
