# Supplementary material for: Secretor and non-secretor human milk oligosaccharides differentially modulate immune response in the presence of cow’s milk allergen β-lactoglobulin in an in vitro sensitization model
Source: Front Immunol. 2025 May 9;16:1575656. doi: 10.3389/fimmu.2025.1575656 (PMC12098339; doi:10.3389/fimmu.2025.1575656)
Supplement: Supplementary file 1 [file DataSheet1.docx]

| **Table S1**. Levels of human milk oligosaccharides in secretor (Se+) and non-secretor (Se-) pooled human milk samples after endotoxin removal at highest concentration used in experiments (0.5%; 5mg/ml) | | | | |
| --- | --- | --- | --- | --- |
|  | Se+ pooled HMOs | | Se- pooled HMOs | |
|  | µg/ml | Recovery | µg/ml | Recovery |
| 3-FL | 137 | 93% | 93.9 | 93% |
| 2’-FL | 324 | 95% | 17.4 | 80% |
| LNT | 355 | 94% | 737 | 92% |
| 6’-SL | 84.0 | 93% | 74.3 | 90% |
| 3’-SL | 60.6 | 93% | 32.2 | 91% |
| HMOs: human milk oligosaccharides, 3-FL: 3-Fucosyllactose, 2’-FL: 2’-Fucosyllactose, LNT: Lacto-N-tetraose, 6’-SL: 6’-Sialyllactose, 3’-SL: 3’-Sialyllactose. | | | | |

**Figure S1.** Effects of ovalbumin and cow’s milk allergen(fractions) whey, β-lactoglobulin (BLG), casein and αs-casein exposed for 48h to moDCs with or without HT-29 (IEC) on basolateral cytokine concentration and surface marker expression of CD14-/HLA-DR+/CD209+ moDCs. Data is presented as mean ± standard error of the mean. Differences between interventions with control were tested by repeated measures one-way ANOVA with Dunnett’s multiple comparison posthoc test or Friedman with Dunn’s multiple comparison posthoc test. *p≤0.05.

**Figure S2.** Effects of non-secretor (Se-) and secretor (Se+) pHMOs with β-lactoglobulin (BLG) on moDCs with or without HT-29 (IEC) on basolateral supernatant cytokine concentration and surface marker expression of CD14-/HLA-DR+/CD209+ moDCs. Data is presented as mean ± standard error of the mean. Differences between interventions with BLG were tested by repeated measures one-way ANOVA with Dunnet’s posthoc test or Friedman test. *p≤0.05, **p ≤0.01.

**Figure S3.** Effects of moDCs exposure to non-secretor (Se-) and secretor (Se+) pHMOs with β-lactoglobulin (BLG) in the presence of HT-29 (IEC) on supernatant cytokine concentration and surface marker expression of CD4+/CD25+/FoxP3+/CD45RA-/CD45RO+ T cells. Data is presented as mean ± standard error of the mean. Differences between interventions with BLG were tested by repeated measure one-way ANOVA with Dunnet’s multiple comparison posthoc test or Friedman test with Dunns multiple comparison posthoc test. *p≤0.05.

**Figure S4.** Effects of moDCs with or without HT-29 (IEC) exposure to non-secretor (Se-) and secretor (Se+) pHMOs with β-lactoglobulin (BLG) on supernatant cytokine concentration and surface marker expression of CD4+/CD25+/FoxP3+/CD45RA-/CD45RO+ T cells. Data is presented as mean ± standard error of the mean. Differences between interventions with BLG were tested by repeated measure one-way ANOVA with Dunnet’s multiple comparison posthoc test or Friedman test with Dunns multiple comparison posthoc test. *p≤0.05.
